# ﻿Redescription of *Malacosarcusmacrostoma* (Günther, 1878) from the abyssal zone off Taiwan, northwestern Pacific Ocean (Beryciformes, Stephanoberycoidei, Stephanoberycidae)

**DOI:** 10.3897/zookeys.1220.126580

**Published:** 2024-12-09

**Authors:** Yo Su, Mao-Ying Lee, Hsuan-Ching Ho

**Affiliations:** 1 Department and Graduate Institute of Aquaculture, National Kaohsiung University of Science Technology, Kaohsiung, Taiwan National Kaohsiung University of Science Technology Kaohsiung Taiwan; 2 Marine Fisheries Division, Fisheries Research Institute, Ministry of Agriculture, Executive Yuen, Keelung, Taiwan Fisheries Research Institute, Ministry of Agriculture Keelung Taiwan; 3 Australian Museum, Sydney, Australia Australian Museum Sydney Australia

**Keywords:** Biodiversity, biogeography, deep sea, ichthyology, taxonomy

## Abstract

The large-mouth pricklefish, *Malacosarcusmacrostoma* (Günther, 1878), previously known from five specimens collected from the central Pacific Ocean, is redescribed based on three specimens collected from the abyssal zone (4,412–4,813 m) off the southeastern coast of Taiwan. These specimens contribute to a more comprehensive description of *M.macrostoma* and represent this species’ westernmost and deepest record. This study provides a detailed description, fresh photographs, and notes on their morphological characteristics of the specimens. Lastly, the distributional records of *M.macrostoma* are discussed.

## ﻿Introduction

The pricklefish family Stephanoberycidae comprises benthopelagic or abyssal-benthic fishes, which can be found worldwide reaching depths down to 5,300 m (Kotlyar 1996; Moore 2003, 2016). They are characterized by having a single dorsal fin positioned posteriorly, symmetrical with the anal fin, 0–3 weak spines and 9–14 soft rays on dorsal and anal fin, 0 spines and 5–6 soft rays on the pelvic fin, 9–12 procurrent caudal-fin rays, no teeth on the vomer and palatine, minute spines on bony ridges of the head, and a single supramaxilla (Moore 2003, 2016).

Presently, four species, each belonging to distinct monotypic genera, are recognized as valid: *Abyssoberyxlevisquamosus* Merrett & Moore, 2005, *Acanthochaenusluetkenii* Gill, 1884, *Malacosarcusmacrostoma* (Günther, 1878), and *Stephanoberyxmonae* Gill, 1883 (Fricke et al. 2024). Among these, *M.macrostoma* was originally described from the tropical mid-Pacific region based on a specimen (Günther 1878). However, specimens of this species appear to be extremely rare in collections worldwide, which were known from five specimens collected from sparse localities in the central Pacific Ocean (Günther 1878; Boehlert and Mundy 1992; Nonaka et al. 2021). Moreover, adult specimens are either disintegrated or in poor condition, including the fragmented holotype (Merrett and Moore 2005; J. Maclain pers. comm. 11 Apr. 2024). Currently, the available specimens of *M.macrostoma* are predominantly in the larval stage (Nonaka et al. 2021). Therefore, information regarding the biology, ecology, and detailed morphology remains incomplete.

During a revision of stephanoberycoid fishes in Taiwan, three specimens collected by R/V *Ocean Researcher I* from the abyssal zone (4,412–4,813 m) off southeastern Taiwan were discovered in the collection of the Biodiversity Research Center, Academia Sinica, Taiwan (ASIZP). Upon detailed examination, these specimens were confirmed to be *Malacosarcusmacrostoma* based on diagnostic characteristics. Consequently, these specimens represent both the westernmost record and the first record from Taiwan and the northwestern Pacific Ocean.

Given the importance of establishing a clear taxonomic status for *M.macrostoma*, a species reported based on limited specimens, this study aims to provide a precise, specimen-based description, fresh photographs, morphological characters, and distribution records of *M.macrostoma*.

## ﻿Materials and methods

The specimens were fixed in 95% EtOH and permanently preserved in 70% EtOH. They are deposited at Biodiversity Research Center, Academia Sinica, Taipei, Taiwan (**ASIZP**). The distribution map was generated using Ocean Data View (Schlitzer 2024).

Terminology and methodology follow Kotlyar (1996) and Su et al. (2023a). Gill rakers were counted on the outer faces of all four arches and abbreviated as GRI–IV. Body depth was measured at the greatest depth. Counts of paired-fin characters were presented as left/right whenever available.

Measurements were made using 150 mm digital calipers under a stereomicroscope (Olympus SZ51) and rounded to the nearest 0.1 mm. Morphometric data were expressed as percentages or ratios of standard length (**SL**) and/or head length (**HL**), except otherwise indicated.

## ﻿Results

**Family Stephanoberycidae Gill, 1884**

### ﻿ Malacosarcus macrostoma

Taxon classificationAnimaliaStephanoberyciformesStephanoberycidae

(Günther, 1878)

3B41D9A4-8732-5FC8-A1E6-8EB245E95708

**Figure 1.**
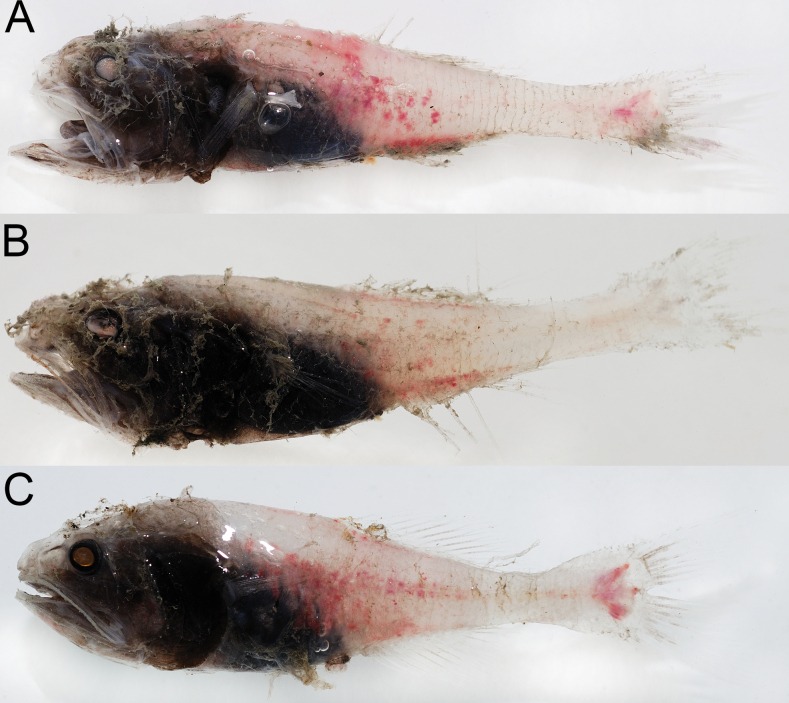
Fresh specimens of *Malacosarcusmacrostoma* (Günther, 1878) **A**ASIZP 73637, 65.5 mm SL, left-right reversed **B**ASIZP 73644, 55.7 mm SL, left-right reversed **C**ASIZP 73646, 61.2 mm SL.

**Figure 2.**
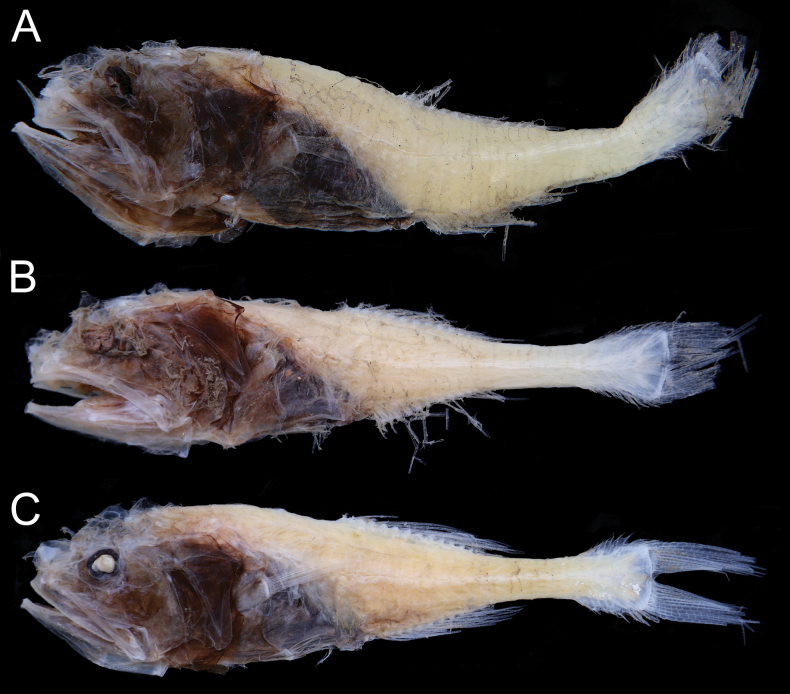
Preserved specimens of *Malacosarcusmacrostoma* (Günther, 1878) **A**ASIZP 73637, 65.5 mm SL**B**ASIZP 73644, 55.7 mm SL**C**ASIZP 73646, 61.2 mm SL. Photos by Y.-C. Hsu.

**Figure 3.**
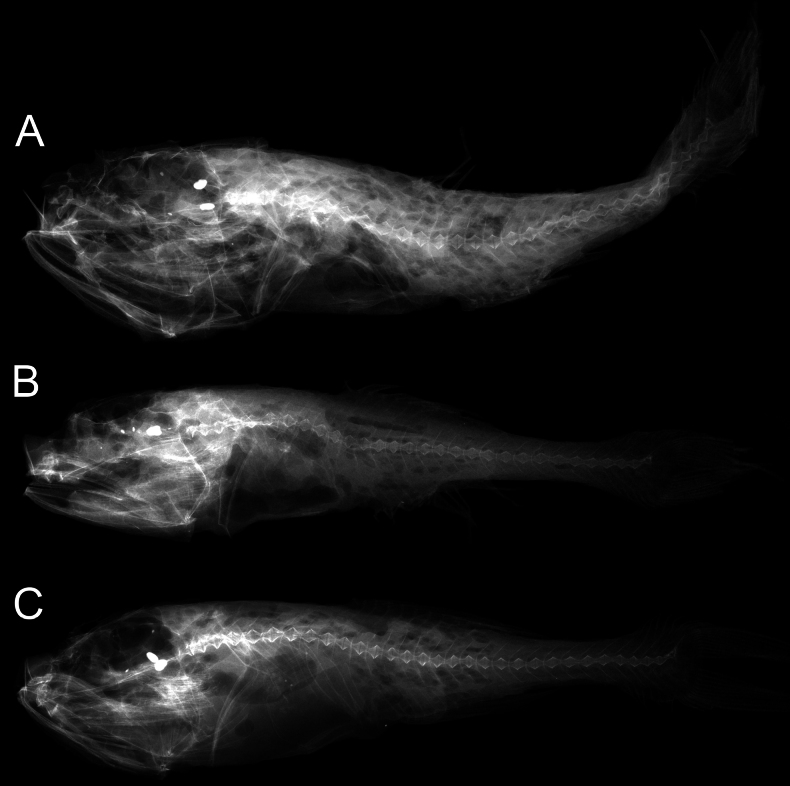
X-radiographs of *Malacosarcusmacrostoma* (Günther, 1878) **A**ASIZP 73637, 65.5 mm SL**B**ASIZP 73644, 55.7 mm SL**C**ASIZP 73646, 61.2 mm SL.

Figs 1 , 2 , 3 , Tables 1 , 2 English name: Large-mouth pricklefish Chinese name: 大口軟冠鯛

Scopelus macrostoma Günther, 1878: 186 (type locality: Mid Pacific, 0°33'S, 154°34'W, depth 2,425 fathoms (4,435 m). Holotype: BMNH 1887.12.7.11).
Malacosarcus macrostoma : Günther 1887: 30 (new genus and new combination). Goode and Bean 1896: 182 (in part). Grey 1956: 191 (listed). Ebeling and Weed 1973: 416 (in part). Kotlyar 1996: 261 (in part). Kotlyar 2004: 2 (listed). Merrett and Moore 2005: 1708 (in part, compared to the new genus and species described). Mundy 2005: 301 (listed, Hawaiian Islands). Nonaka et al. 2021: 153, fig. 9B (in part, larval record from the Hawaiian Islands).
Stephanoberycidae sp.: Boehlert and Mundy 1992: 83, fig. 4 (larval record from Hancock Seamount, Hawaiian Islands).

#### Specimens examined.

Taiwan • ASIZP 73637, 65.5 mm SL, off southeastern Taiwan, Philippine Sea, 22°3'38.16"N, 121°10'6.95"E, R/V Ocean Researcher I, sta. CP 413, beam trawl, depth 4,412–4,458 m, 12 Jun. 2008; ASIZP 73644, 55.7 mm SL, off southeastern Taiwan, Philippine Sea, 22°7'32.15"N, 122°5'35.87"E, R/V Ocean Researcher I, sta. CP 415, beam trawl, depth 4,806–4,813 m, 14 Jun. 2008; ASIZP 73646, 61.2 mm SL, off southeastern Taiwan, Philippine Sea, 22°12'28.07"N, 122°6'56.16"E, R/V Ocean Researcher I, sta. CP 415, beam trawl, depth 4,801–4,813 m, 15 Jun. 2008. All collected by M.-Y. Lee.

#### Diagnosis.

A species of Stephanoberycidae characterized by the absence of dorsal-and anal fin-spines (vs fin spines present in other genera, sometimes absent in *Acanthochaenus*); 11–12 anal-fin rays (vs 9–10 in *Acanthochaenus*); GRI 7–8 + 1 + 15–19 = 23–27 (vs 5–7 + 1 + 13–14 = 19–21 in *Abyssoberyx* and 12–15 + 1 + 24–27 = 37–40 in *Stephanoberyx*); lateral line forms flaps in approximately 42–46 vertical rows (vs lateral line without distinct vertical flaps in *Acanthochaenus* and *Stephanoberyx*); vertebrae 10 + 20–21 = 30–31 (vs 10–11 + 21–23 = 32–34 in *Abyssoberyx*); body scales cycloid and deciduous (vs body scales ctenoid and adherent in *Acanthochaenus* and *Stephanoberyx*); and origin of pelvic fin situated nearer to pectoral fin than to anal fin (vs pelvic fin situated nearer to anal fin than to pectoral fin in *Acanthochaenus*).

#### Description of Taiwanese specimens.

Meristic and morphometric data are provided in Tables 1 and 2.

**Table 1.** Meristic characters of *Malacosarcusmacrostoma* (Günther, 1878). Paired-fin characters are presented as left/right whenever available. Abbreviations: GRI–IV = gill rakers on first to fourth arches.

	ASIZP 73637	ASIZP 73644	ASIZP 73646
Dorsal-fin rays	13	13	14
Pectoral-fin rays	13/12	12/13	12/13
Pelvic-fin rays	6/5	5/5	4/4
Anal-fin rays	11	12	11
Caudal-fin rays	N/A	9+10+9+10	9+10+9+11
GRI	8+1+16=25	7+1+15=23	8+1+18=27
GRII	6+1+17=24	6+1+15=22	7+1+17=25
GRIII	3+1+14=18	3+1+14=18	3+1+14=18
GRIV	1+1+10=12	2+0+11=13	1+1+12=14
Pseudobranchial filaments	N/A	5	6
Lateral-line flaps	ca 46	N/A	ca 42
Vertebrae	10+20=30	10+20=30	10+21=31

**Table 2.** Morphometric characters of *Malacosarcusmacrostoma* (Günther, 1878). Abbreviations: A = anal-fin; C = caudal-fin; D = dorsal-fin; HL = head length; N/A = not available; P = pectoral-fin; SL = standard length; V = pelvic-fin.

	ASIZP 73637	ASIZP 73644	ASIZP 73646
SL (mm)	65.5	55.7	61.2
% SL			
HL	35.8	36.0	37.5
Head depth	24.2	23.1	28.9
Predorsal length	51.1	49.4	50.2
Prepectoral length	38.2	39.7	40.5
Prepelvic length	45.8	40.3	44.5
Preanal length	59.7	51.3	58.1
Snout length	7.8	9.1	9.0
Eye diameter	7.6	7.3	8.2
Interorbital width	12.5	N/A	12.2
Forehead height	5.9	N/A	5.1
Postorbital length	19.0	21.5	22.0
Upper-jaw length	N/A	24.9	24.0
Lower-jaw length	25.6	26.3	27.0
D–P length	18.0	14.0	16.2
D–V length	19.8	18.9	24.9
Greatest body depth	23.5	23.4	24.4
P length	14.2	N/A	16.3
V length	7.1	N/A	N/A
P–V length	9.2	9.3	9.5
D–A length	19.8	18.3	22.5
V–A length	15.4	12.4	16.1
D length	23.4	24.3	24.8
A length	17.9	18.2	19.6
Postanal length	28.0	26.9	28.1
Postdorsal length	25.8	24.5	27.4
C length	N/A	N/A	23.8
Caudal-peduncle height	7.0	6.3	6.8
Longest gill raker	8.1	7.8	9.5
Gill filament at angle	1.0	0.5	0.8
%HL			
Head depth	67.6	64.1	77.1
Snout length	21.9	25.4	24.0
Eye diameter	21.3	20.4	21.8
Interorbital width	35.0	N/A	32.5
Forehead height	16.4	N/A	13.7
Postorbital length	53.1	59.7	58.8
Upper-jaw length	N/A	69.2	64.0
Lower-jaw length	71.5	73.2	72.0

Dorsal-fin rays 13–14. Pectoral-fin rays 12–13/12–13. Pelvic-fin rays 4–6/4–5. Anal-fin rays 11–12. Principal caudal-fin rays 10 + 9, uppermost and lowermost rays unbranched; procurrent caudal-fin rays 9 and 10–11 on upper and lower lobes, respectively. GRI 7–8 + 1 + 15–18 = 23–27 (total); GRII 6–7 + 1 + 15–17 = 22–25; GRIII 3 + 1 + 14 = 18; GRIV 1–2 + 0–1 + 10–12 = 12–14. Pseudobranchial filaments 5–6 (*n* = 2). Lateral-line flaps in approximately 42–46 vertical rows. Vertebrae 10 + 20–21 = 30–31; branchiostegal rays 8.

Body slender, greatest depth 4.1–4.3 in SL; body laterally compressed. Head oval, length 2.7–2.8 in SL; its height 1.3–1.6 in HL; upper profile of head slightly rounded, with nearly straight profile to dorsal-fin origin; forehead slightly convex, its height 6.1–7.3 in HL; eye diameter 4.6–4.9 in HL; tip of snout rounded, extending slightly before premaxilla, its length 3.9–4.6 in HL; interorbital width 2.9–3.1 in HL.

Mouth oblique, upper-jaw length 1.4–1.6 in HL; posterior end of maxilla rounded, exceeding beyond vertical through posterior margin of eye; lower jaw slightly larger than upper jaw, length 1.4 in HL; its anterior tip protruding before upper jaw when closed. Two nostrils situated in front of eye; both at same horizontal through center of eye; both nostrils rounded and nearly same in size. No trace of nasal organs in all specimens (possibly shrunk during preservation). Symphysis of premaxillae notched and naked. Symphysis of dentaries forming single and blunt knob. Supramaxilla single, with needle-like process anteriorly and rectangular, slightly oval process posteriorly; its posterior tip in advance of tip of maxilla.

Head skeletons and their ridges delicate. Opercle with one central ridge but not forming spine. Small spines on posteroventral margins of both inner and outer ridges of preopercle. Posttemporal ridge rounded, with small spines on its outer margin. Both premaxilla and dentary with villiform teeth on outer and medial surfaces. Palatine and vomer edentate.

Gill rakers on outer face of all four arches rod-shaped and laterally compressed, their inner surfaces covered with small teeth; rakers on outer row of first arch longer than remainder, longest gill raker in 4.0–4.6 in HL; rakers on inner surfaces of outer three arches absent or forming minute bumps; no tooth patches present between rakers on all four arches. Fourth gill arch largely attached to the wall leaving slit between arch and gill chamber. No tooth on fifth ceratobranchial. Oval, conical tooth patch on second epibranchial arch. Large, moon-shaped conical tooth patch on fourth pharyngobranchial. Gill filaments present on all four rakers. Gill filaments on first arch very short, 8.4–14.9 in length of longest opposite rakers. Pseudobranch present, short and poorly developed.

Body scales cycloid and deciduous, embedded under skin; those covered by pectoral fin smaller than rest. Lateral-line scales and abdominal scutes absent. No scales on gular region and isthmus. Cycloid scales present on cheek.

Dorsal fin situated posteriorly, its origin about same vertical of anus. Pectoral-fin length 2.3–2.5 in HL; its origin at horizontal through ventral margin of eye; its tip reaching vertical through anus. Pelvic-fin length 5.0 in HL; its origin behind pectoral-fin base and tip reaching anus when adpressed. Anal-fin origin at vertical through fifth dorsal-fin ray; its posterior end at same vertical through that of dorsal fin. Caudal fin moderately small, forked. All fin rays delicate, with smooth surfaces.

Skins on surface of lateral line forming many vertical rows of flaps. Its main branch forming canal, originating behind and below posttemporal bone; its anterior portion gently curved down, becoming nearly straight on posterior portion. Anus situated immediately anterior to anal-fin origin. Caudal peduncle slender, with postdorsal and postanal length 1.4–1.5 and 1.3 in HL, respectively; its height 5.1–5.7 in HL. Light organs absent.

#### Coloration.

When fresh (Fig. 1), body pale and somewhat translucent; head and abdominal region uniformly black; all fins pale; lateral-line flaps forming vertical dark bands on lateral side of body. When preserved (Fig. 2), body and fins uniformly pale; oral cavity, including underside of tongue, inner face of operculum, and gill arches brown; gill rakers pale.

#### Osteology

**(Fig. 3).** Three supraneurals. First dorsal-fin pterygiophore inserts between eighth and ninth or seventh and eighth vertebra. Pleural ribs present on sixth to tenth vertebra; epineurals present. Anal-fin origin below twelfth vertebra.

#### Size.

A rather small species, reported up to 8.9 cm (Günther 1878).

#### Distribution.

**Figure 4.**
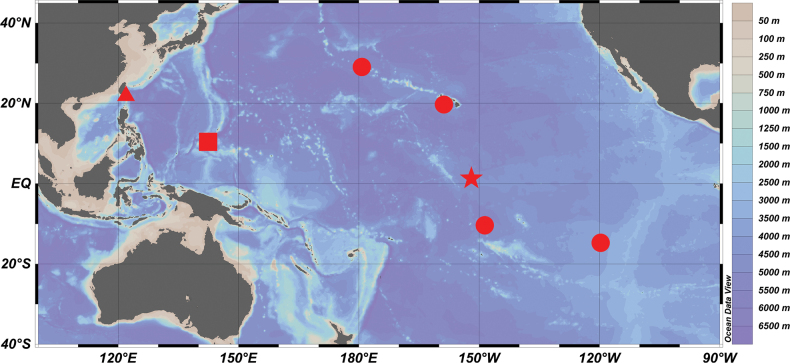
Distribution map of *Malacosarcusmacrostoma* (Günther, 1878). Data source: triangle = this study; star = type locality; circle = other records (Günther 1887; Ebeling and Weed 1973; Boehlert and Mundy 1992; Nonaka et al. 2021); square = possible records (Jamieson et al. 2021).

Known from specimens collected from the tropical central Pacific Ocean at depths 2,777–4,434.8 m (Günther 1878; Ebeling and Weed 1973; Mundy 2005). Our specimens were collected from the northwestern Pacific at depths 4,412–4,813 m (Fig. 4).

## ﻿Discussion

### ﻿Comments on morphological characters

Our specimens were identified as *Malacosarcusmacrostoma* based on several distinguishing features, including the absence of dorsal-fin spines, 7–8 + 1 + 15–18 = 23–27 rakers on the first gill arch, 9–11 procurrent caudal-fin rays, and deciduous and cycloid scales on the body (Kotlyar 1996; Merrett and Moore 2005). Among the four stephanoberycids, *M.macrostoma* is most similar to *Abyssoberyxlevisquamosus*, sharing similar head and body shapes, deciduous and cycloid body scales, and dark vertical bars on the body (Kotlyar 1996; Merrett and Moore 2005). However, *M.macrostoma* can be differentiated from *A.levisquamosus* by the absence of dorsal- and anal-fin spines (vs one dorsal-fin spine and one or two anal-fin spines in *A.levisquamosus*; Merrett and Moore 2005), 7–8 + 1 + 15–18 = 23–27 gill rakers (vs 5–7 + 1 + 13–14), 10 + 20–21 = 30–31 vertebrae (vs 10–11 + 21–23 = 32–34), and pectoral-fin origin at horizontal ventral margin of eye (vs distinctly below).

All of our specimens exhibit dark vertical flaps along the lateral side of the body, similar to those of *A.levisquamosus* (Merrett and Moore 2005). While Merrett and Moore (2005) identified these structures as scale pockets, we propose that they are fused vertical papillae formed by the lateral-line system, akin to those observed in *Rondeletia* (Paxton 1999) and *Gibberichthys* (de Sylva and Eschmeyer 1977; Ho et al. 2023), based on our observation that the body scales are much smaller than the width of these vertical flaps.

In this study, we did not find either nasal organs or Tominaga’s organ (sensu Paxton et al. 2001) in the nasal chamber of all specimens. However, nasal organs are present in other stephanoberycoid taxa, such as *Rondeletialoricata* Abe & Hotta, 1963, *Gibberichthyspumilus* Parr, 1933, *Hispidoberyxambagiosus* Kotlyar, 1981, and cetomimids (Paxton 1989; Paxton et al. 2001; Su et al. 2023b). Therefore, there is a possibility that they deteriorated during the preservation process.

### ﻿Possible records of *M.macrostoma*

In their description of *Abyssoberyxlevisquamosus*, Merrett and Moore (2005) noted the Hawaiian record of *M.macrostoma* (Boehlert and Mundy 1992) may represent their new species. Additionally, they mentioned that the specimen has dorsal- and anal-fin rays and vertebrae differing by one or two counts from the Atlantic specimens of *A.levisquamosus*. Since the diagnostic characters (i.e., numbers of dorsal- and anal-fin spines, gill rakers, and vertebrae) used to distinguish *M.macrostoma* from *A.levisquamosus* are difficult to observe and determine in subadult or larval specimens, and the total counts of fin rays and vertebrae of our specimens are one or two elements lesser than those of *A.levisquamosus*, the Hawaiian record may represent *M.macrostoma*.

Previous records of *M.macrostoma* from the northeastern Atlantic (Merrett and Marshall 1981; Merrett 1992) were re-identified as a possible specimen of *A.levisquamosus* by Merrett and Moore (2005). Therefore, the occurrence of *M.macrostoma* in Atlantic Ocean is pending till other materials become available.

Another photograph taken at the Mariana Trench (northwestern Pacific Ocean) at a depth of 5,961 m by Jamieson et al. (2021: fig. 3k), was identified as “aff. *Abyssoberyx*”. Nevertheless, judging by the distribution of all stephanoberycids, we lean toward the individual being *M.macrostoma*.

### ﻿Ontogenetic migration

Ontogenetic migration to deeper depths has been observed in other stephanoberycids. For example, larvae of *Acanthochaenusluetkenii* were collected at a depth of 30 m (Kotlyar and Evseenko 1989), while larger or adult specimens were collected at depths of 1,183–5,400 m (Moore et al. 2003; Mincarone et al. 2014). To date, adult specimens of *M.macrostoma* have been collected at depths of 2,777–4,813 m (Ebeling and Weed 1973; this study), and a juvenile specimen was observed and collected via blackwater diving (Nonaka et al. 2021). Based on this information, we suggest that an ontogenetic migration to deeper depths occurs in *M.macrostoma*.

## Supplementary Material

XML Treatment for Malacosarcus macrostoma

## ﻿Additional information

### Author ORCIDs

Yo Su https://orcid.org/0000-0002-3576-9229

Mao-Yin Lee https://orcid.org/0000-0002-4643-3173

Hsuan-Ching Ho https://orcid.org/0000-0003-1154-601X

### Data availability

All of the data that support the findings of this study are available in the main text.
